# Illusory competence vs. real needs a cross-sectional study on the mismatch in Smart Senior Care (SSC) perceptions between nursing students and older adults

**DOI:** 10.3389/fpubh.2026.1777839

**Published:** 2026-04-02

**Authors:** Changfu Chen, Miao Zhan, Jiali Zhou, Hongying Zhang

**Affiliations:** 1 The Affiliated Rehabilitation Hospital of Chongqing Medical University, Chongqing, China; 2 The Second Affiliated Chongqing Medical University, Chongqing, China

**Keywords:** needs assessment, nursing education, older adults, perception gap, privacy, smart senior care

## Abstract

**Background:**

Smart Senior Care (SSC) is vital for aging populations, yet its success depends on aligning end-users’ (older adults) needs with future providers’ (nursing students) perceptions. Limited research directly compares these groups to reveal supply–demand discrepancies.

**Objectives:**

To compare self-perceived SEC knowledge, privacy concerns, and service preferences between community-dwelling older adults and nursing students, and to identify supply–demand misalignments relevant to education and service design.

**Methods:**

A comparative cross-sectional survey was conducted in Chongqing, China (June–September 2025) using convenience sampling (older adults, *n* = 280; nursing students, *n* = 171). A Delphi-validated questionnaire assessed perceived SEC knowledge, privacy concerns, and preferences across five SEC domains. Group differences were tested using Mann–Whitney U and chi-square tests. Logistic regression examined predictors of students’ intention to work in SEC. Economic pathways linking income, satisfaction, and willingness-to-pay among older adults were explored using Sankey analysis.

**Results:**

Nursing students reported higher perceived SEC knowledge and lower privacy concern than older adults (both *p* < 0.001). Students prioritized technology- and monitoring-oriented services (e.g., health monitoring, medical nursing), whereas older adults more often emphasized daily-life assistance and emotional support. Educational intervention was the strongest predictor of students’ career intention in SEC (OR = 2.85, 95% CI 1.45–5.60, *p* < 0.05). Lower service satisfaction was consistently associated with lower willingness-to-pay across income groups.

**Conclusion:**

Substantial perception gaps exist between future providers and older adults regarding SEC, particularly around privacy and non-medical needs. Integrating user-centered, community-based training and digital ethics into nursing curricula, while prioritizing service quality and trust-building in SEC design, may improve adoption and sustainability.

## 1 Introduction

Population aging has evolved into a pervasive global demographic challenge, placing unprecedented strain on traditional healthcare systems and necessitating innovative service delivery models (1). In this context, Smart Senior Care (SSC)—the strategic integration of Internet of Things (IoT), big data analytics, and artificial intelligence into geriatric nursing—has emerged as a promising approach to alleviate care resource shortages and support the paradigm of “Aging in Place” (2). Recent systematic reviews indicate that SEC technologies, such as fall detection sensors and medication management robots, may reduce hospital readmission rates and alleviate caregiver burden (3–5). By deploying technologies ranging from wearable physiological monitors to intelligent emergency response systems, SEC aims to enhance the safety, autonomy, and quality of life for the aging population (6). Consequently, governments in rapidly aging societies, particularly China, have elevated the digitalization of care services for people over 65 to a national strategic priority (7).

However, despite the proliferation of technological innovations, the adoption of SEC services in community settings remains limited (8). This gap is often related to the digital divide among older adults, where technology design and user interfaces may not sufficiently accommodate age-related sensory and cognitive changes (9, 10). These challenges suggest that the availability of advanced technology alone does not ensure effective integration into daily life, and highlight the importance of aligning service design with user acceptance and care needs (11). A fundamental challenge lies in bridging the potential “knowledge–practice gap” between SEC design and delivery and its actual utility and acceptance by end-users (12). The implementation of SEC depends on both end-user uptake (older adults) and the capacity of service providers (nurses and medical professionals) to recommend and operate related services (13). Nurses, as key intermediaries within the healthcare system, can influence how complex technologies are communicated and applied in care for older adults (14). Yet, current research has often examined these two stakeholder groups separately. Studies have applied frameworks such as the Technology Acceptance Model (TAM) to explore older adults’ privacy concerns and resistance to change (15–18), while other work has focused on nursing students’ digital literacy and attitudes toward e-health (19). However, technical proficiency does not necessarily translate into user-centered care. Evidence suggests that an excessive emphasis on technical skills in curricula may leave less attention to humanistic values and communication competencies, which may affect person-centered practice (20). Importantly, comparative research directly examining these two groups within the same context remains limited. As nursing students represent the future workforce and are trained in increasingly technology-rich environments (21), it is unclear whether their understanding of “smart care” aligns with older adults’ lived experiences and practical preferences. If future professionals place disproportionate emphasis on technology-intensive monitoring while giving less attention to psychosocial and daily living support (22), a mismatch between service supply and user needs may occur, potentially affecting service relevance and long-term sustainability (23).

To address this gap, this study conducted a comparative cross-sectional investigation between community-dwelling older adults and nursing students. The primary objectives were threefold: (1) to examine disparities in self-perceived knowledge levels and data privacy concerns regarding SEC between the two groups; (2) to analyze differences in service need preferences, particularly comparing medical monitoring with daily life assistance and mental support; and (3) to identify determinants of nursing students’ willingness to engage in SEC careers and to assess the economic viability of SEC from older adults’ perspectives, with particular attention to the relationship between service satisfaction and willingness-to-pay. By identifying these discrepancies, this study seeks to provide empirical evidence for strengthening nursing education to support more user-centered practice (24). Furthermore, the findings aim to inform policymakers and developers in optimizing SEC service design and economic models to better reflect the needs of the aging population, while remaining ethically appropriate and sustainable (25).

## 2 Methods

### 2.1 Study design

This study employed a comparative cross-sectional design and was conducted from June to September 2025 in Chongqing, China. To accommodate differences in digital literacy and feasibility, data were collected using face-to-face interviews for older adults and an online survey platform for nursing students. Ethical approval was obtained from the Institutional Review Board of Chongqing Rehabilitation Hospital (Approval No.: 2025-31). All participants provided informed consent prior to participation.

### 2.2 Participants

Two populations were recruited using convenience sampling: community-dwelling older adults (aged ≥60 years) residing in Chongqing, China, and undergraduate nursing students from Chongqing Medical University. After excluding incomplete or invalid questionnaires, the final analytical sample comprised 451 participants, including 280 older adults and 171 nursing students. The estimated mean age of the older adults was 63.34 ± 8.63 years, and that of the nursing students was 21.06 ± 0.57 years.

### 2.3 Instrument development

A structured questionnaire was developed based on a comprehensive literature review and was validated through a two-round Delphi expert consultation (26–28). The Delphi panel included experts in geriatric nursing, medical informatics, and public health, who reviewed the items to ensure content validity. The final questionnaire consisted of four sections: (1) demographics (age, gender, education level, prior exposure to smart care; for students, grade level and internship experience); (2) self-perceived knowledge of Smart Senior Care (SSC), assessed using a 5-point Likert scale (1 = very low to 5 = very high) and recoded for group comparisons; (3) privacy concerns, measured on a 5-point Likert scale (1 = no concern to 5 = very high concern) and recoded for analysis; and (4) service needs and preferences. For service preferences, participants indicated the perceived importance (yes/no) of five expert-validated SEC domains: Health Monitoring, Daily Life Assistance, Medical Nursing, Emergency Rescue, and Mental Support (29–31). Older adults additionally reported willingness-to-pay (WTP) for SEC services, categorized as low (<300 RMB/month), medium (300–1,000 RMB/month), or high (>1,000 RMB/month). Nursing students were asked about their willingness to engage in SEC careers after graduation (“yes/likely” vs. “no/unlikely”), which served as the dependent variable for regression analysis.

### 2.4 Statistical analysis

Data were analyzed using IBM SPSS Statistics version 26.0 (IBM Corp., Armonk, NY). Continuous variables are presented as mean ± SD for approximately normally distributed data or median (IQR) for skewed/ordinal data, and categorical variables as frequencies and percentages. Normality was assessed using the Shapiro–Wilk test and Q–Q plots. Group comparisons were performed using the Mann–Whitney U test for continuous/ordinal variables and Pearson’s chi-square test (χ^2^) for categorical variables (Fisher’s exact test when expected counts were <5). Multivariable binary logistic regression was used to examine factors associated with nursing students’ intention to work in SEC (gender, grade level, internship experience, and receipt of SEC-related educational intervention), with results reported as ORs with 95% CIs. Figures were produced using Python (Plotly) and OriginPro 2024. All tests were two-tailed, and *p* < 0.05 was considered statistically significant.

## 3 Results

### 3.1 Demographic characteristics and the cognitive-digital divide

**Figure 1:**
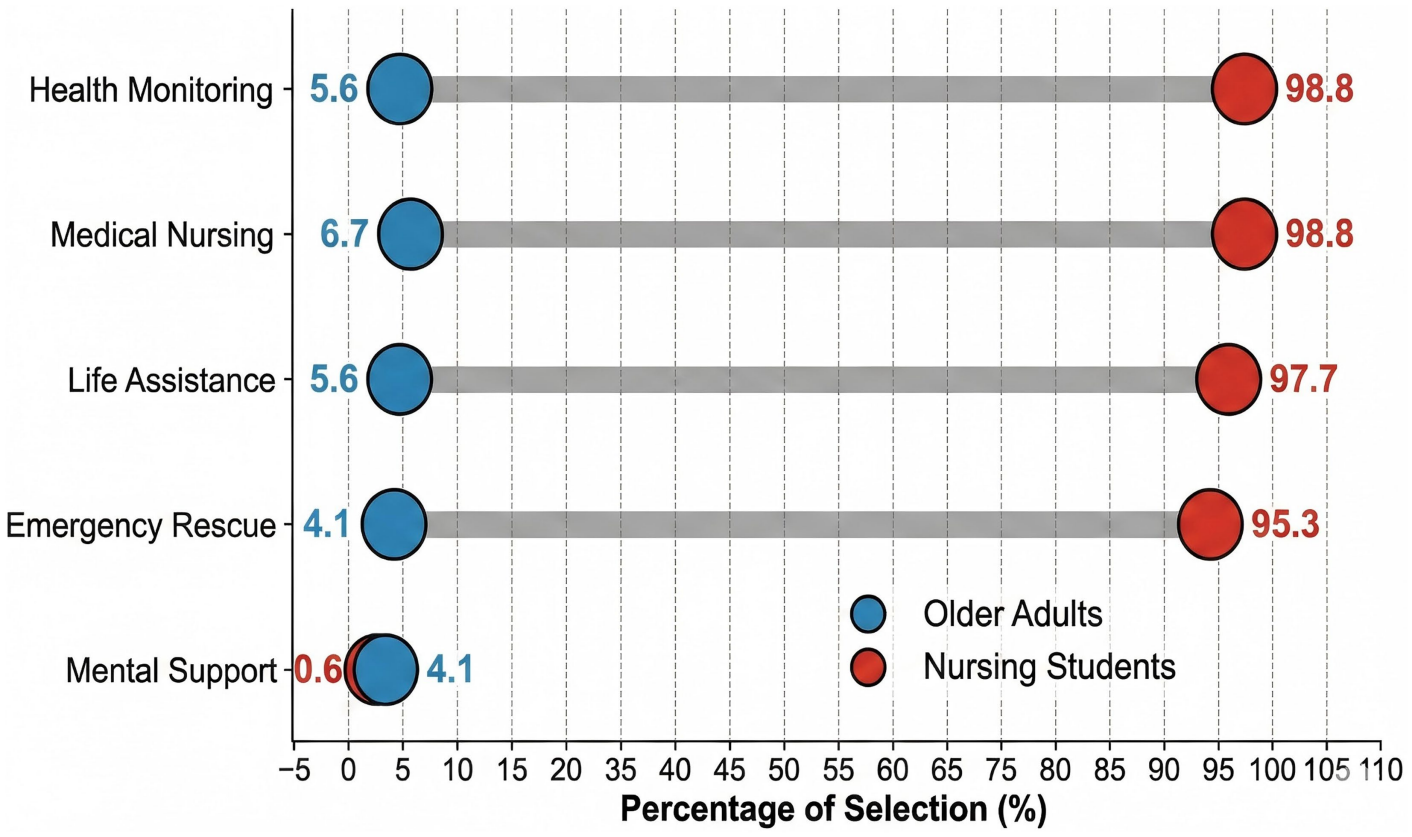
The awareness gap in smart care service categories. Comparison of selection percentages reveals a near-total lack of awareness among older adults (lines near bottom, <7%) contrasted with high awareness among nursing students (lines near top, >95%).

A total of 451 valid questionnaires were analyzed, including 171 nursing students (supply side) and 280 community-dwelling older adults (demand side). The mean age was 21.06 ± 0.57 years among nursing students and 63.34 ± 8.63 years among older adults. Baseline demographic and professional characteristics are summarized in Table 1. Marked differences in prior exposure to Smart Senior Care (SSC) were observed: 76.0% of nursing students reported having received SEC-related training, compared with 16.1% of older adults reporting prior exposure. Nursing students demonstrated higher self-perceived SEC knowledge than older adults (Mann–Whitney U test: *Z* = −5.136, *p* < 0.001) (Table 2). In contrast, older adults reported higher privacy concern scores (*Z* = −5.804, *p* < 0.001). Figure 1 presents the proportions selecting each SEC service domain. Nursing students most frequently selected Health Monitoring and Medical Nursing (both >95%), whereas selection proportions among older adults were lower across domains (4.1–6.7%).

**Table 1:** Demographic characteristics and baseline information of the study population.

Variables	Nursing students (*n* = 171)	Older adults (*n* = 280)
Demographics		
Junior college/year 1–2	20 (11.7)	–
Undergraduate year 3	115 (67.3)	–
Undergraduate year 4	36 (21.0)	–
Age 60–69 years	–	112 (40.0)
Age 70–79 years	–	125 (44.6)
Age ≥ 80 years	–	43 (15.4)
Living alone	–	82 (29.3)
Professional and health background		
With internship experience	59 (34.5)	–
No internship experience	112 (65.5)	–
Self-reported healthy	–	95 (33.9)
Chronic disease (stable)	–	148 (52.9)
Frail/needs assistance	–	37 (13.2)
Smart care readiness		
Education/training received	130 (76.0)	45 (16.1)
Confidence in technology (high/medium)	163 (95.3)	200 (71.4)
Engagement willingness	108 (63.2)	156 (55.7)

**Table 2:** Comparison of knowledge scores and privacy concerns between groups (Mann–Whitney U Test).

Variable	Group	*N*	Mean rank	*Z*	*p*-value
Knowledge score	Older adults	280	201.32	−5.136	<0.001
	Nursing students	171	249.28		
Privacy concern	Older adults	280	243.13	−5.804	<0.001
	Nursing students	171	183.75		

### 3.2 Structural misalignment in service priorities and technology acceptance

**Figure 2:**
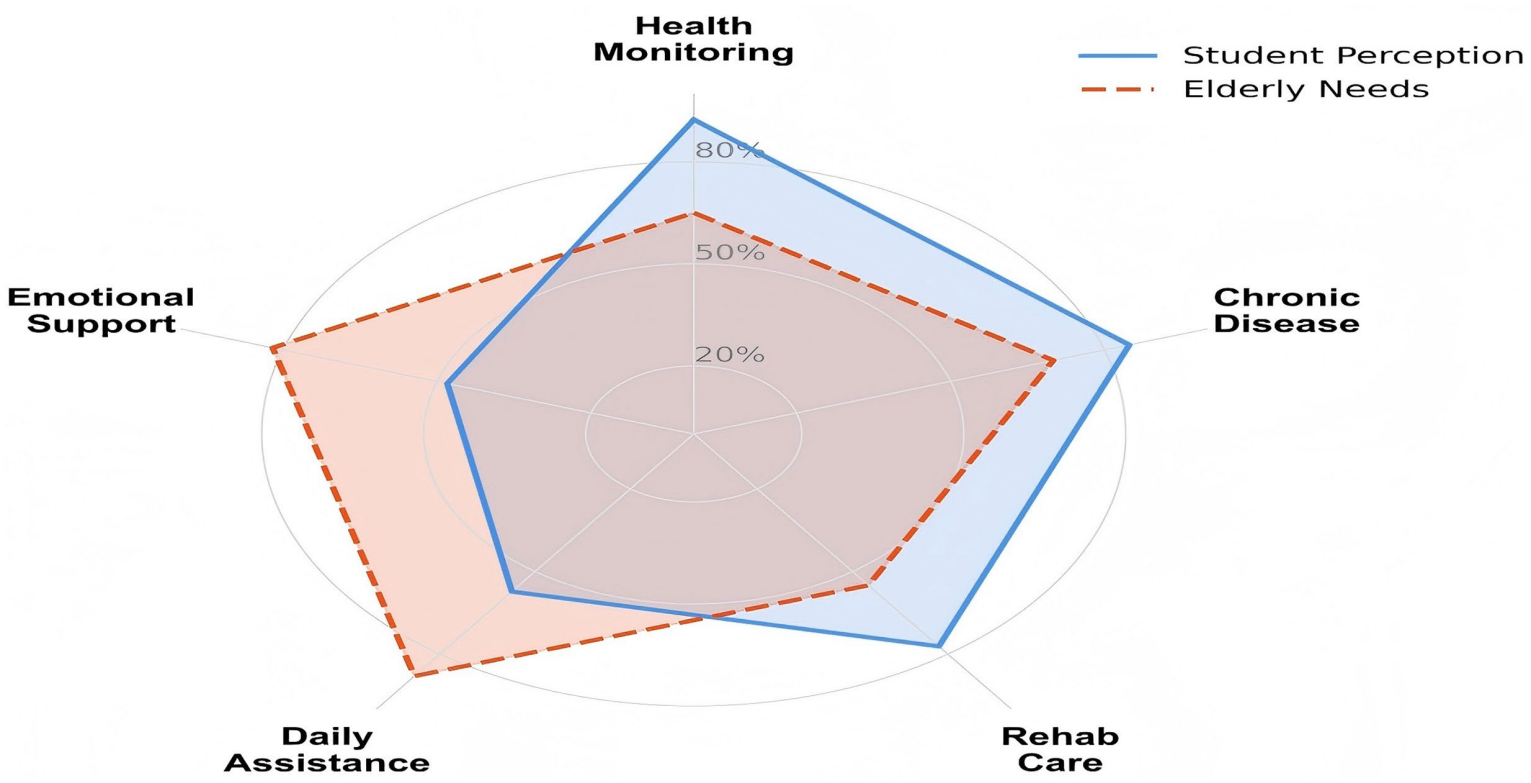
Perceptual divergence in smart care service priorities. The radar chart illustrates the structural mismatch: Students (blue line) overemphasize medical-technical services, while adults over 65 (orange dashed line) show significantly higher prioritized needs for daily assistance and emotional support.

**Figure 3:**
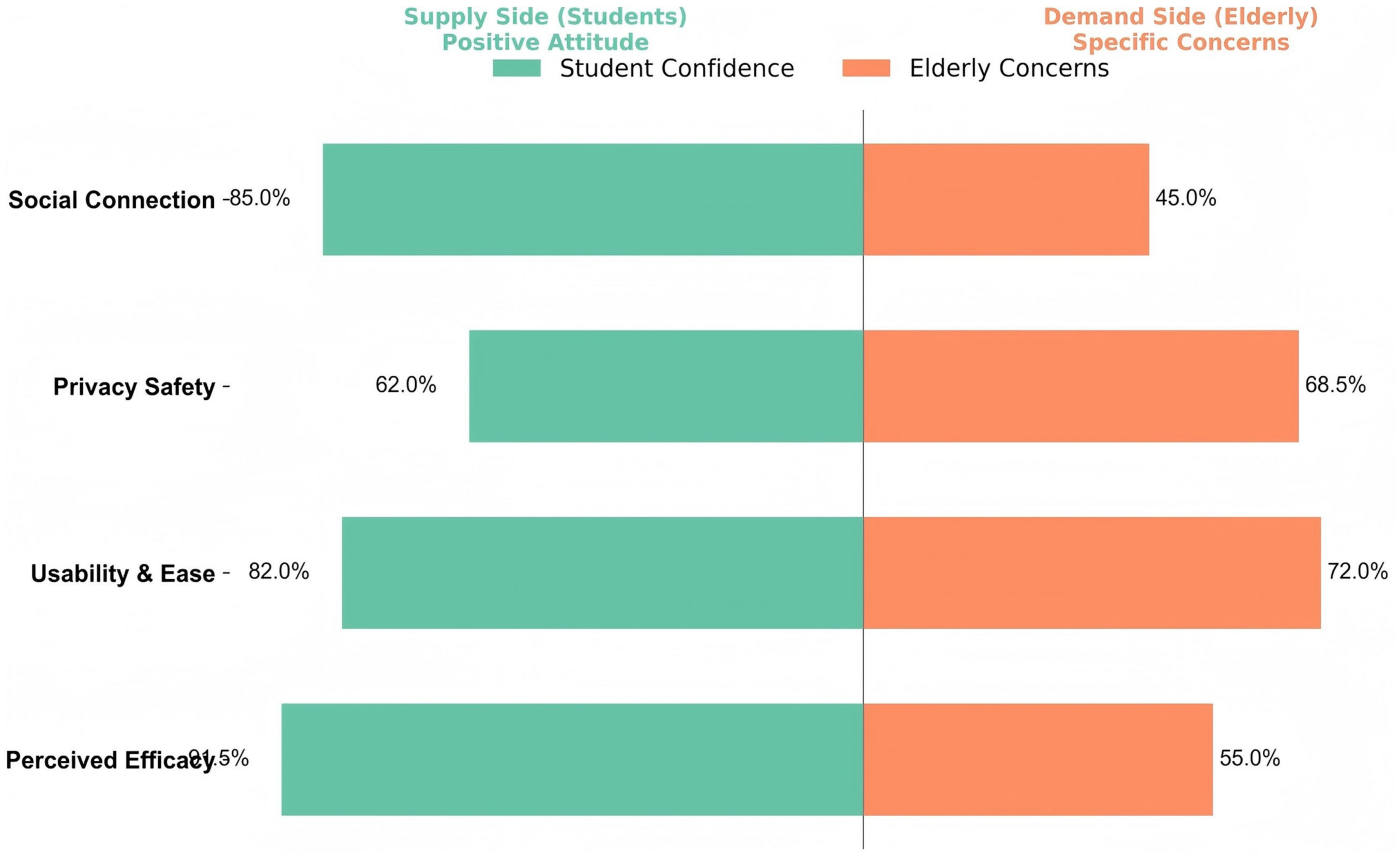
Contrast in attitudes toward technology: Confidence versus Privacy Concerns. The diverging bars reveal a gap between students’ technological optimism (Left) and older adults’ specific concerns regarding usability and privacy (Right).

Beyond the baseline awareness gap, further analysis indicates differences in service priorities and attitudes toward technology between the two groups. As illustrated in the radar chart (Figure 2), nursing students (Blue line) prioritized medical and technology-oriented functions, such as Health Monitoring and Chronic Disease Management, suggesting a stronger focus on clinically oriented aging needs. In contrast, the prioritized needs of participants over the age of 65 (Orange line) were more concentrated on supportive services, particularly Daily Life Assistance and Emotional Support, which were less emphasized by students. This difference in service priorities is accompanied by differences in technology-related attitudes, as shown in the diverging bar chart (Figure 3). On the supply side (Left/Green bars), students reported higher confidence in the effectiveness of SEC and a stronger willingness to recommend these technologies. On the demand side (Right/Orange bars), older adults reported higher levels of concern, especially regarding usability and privacy safety. These patterns suggest that students’ overall optimism about SEC may not fully reflect the user experience barriers reported by older adults, including concerns about complexity and perceptions of surveillance.

### 3.3 Determinants of workforce supply and economic viability

**Figure 4:**
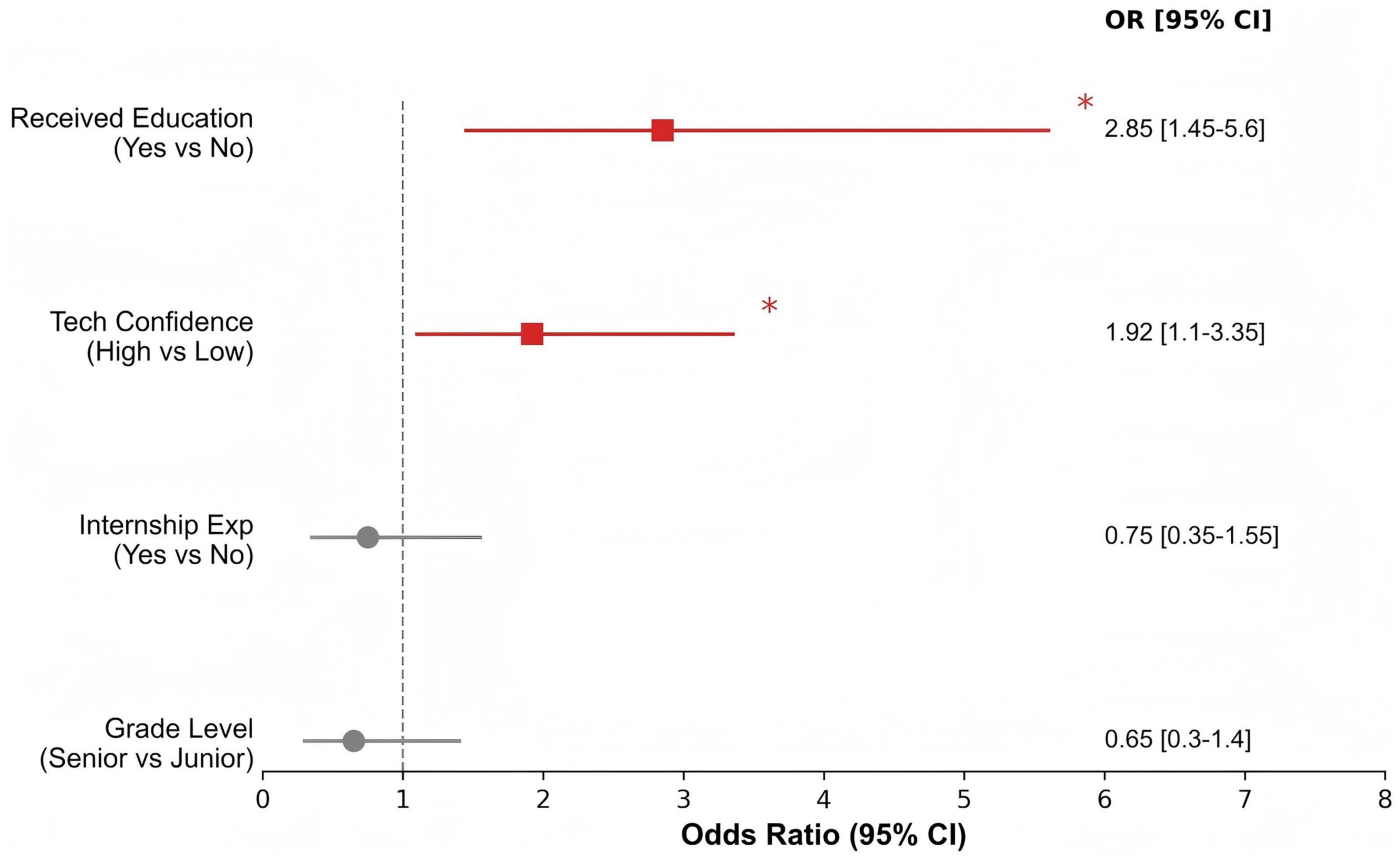
Forest plot of predictors for nursing students’ willingness to work in smart care. Educational intervention is identified as the most significant positive predictor (OR = 2.85). * indicates statistical significance at *p* < 0.05.

**Figure 5:**
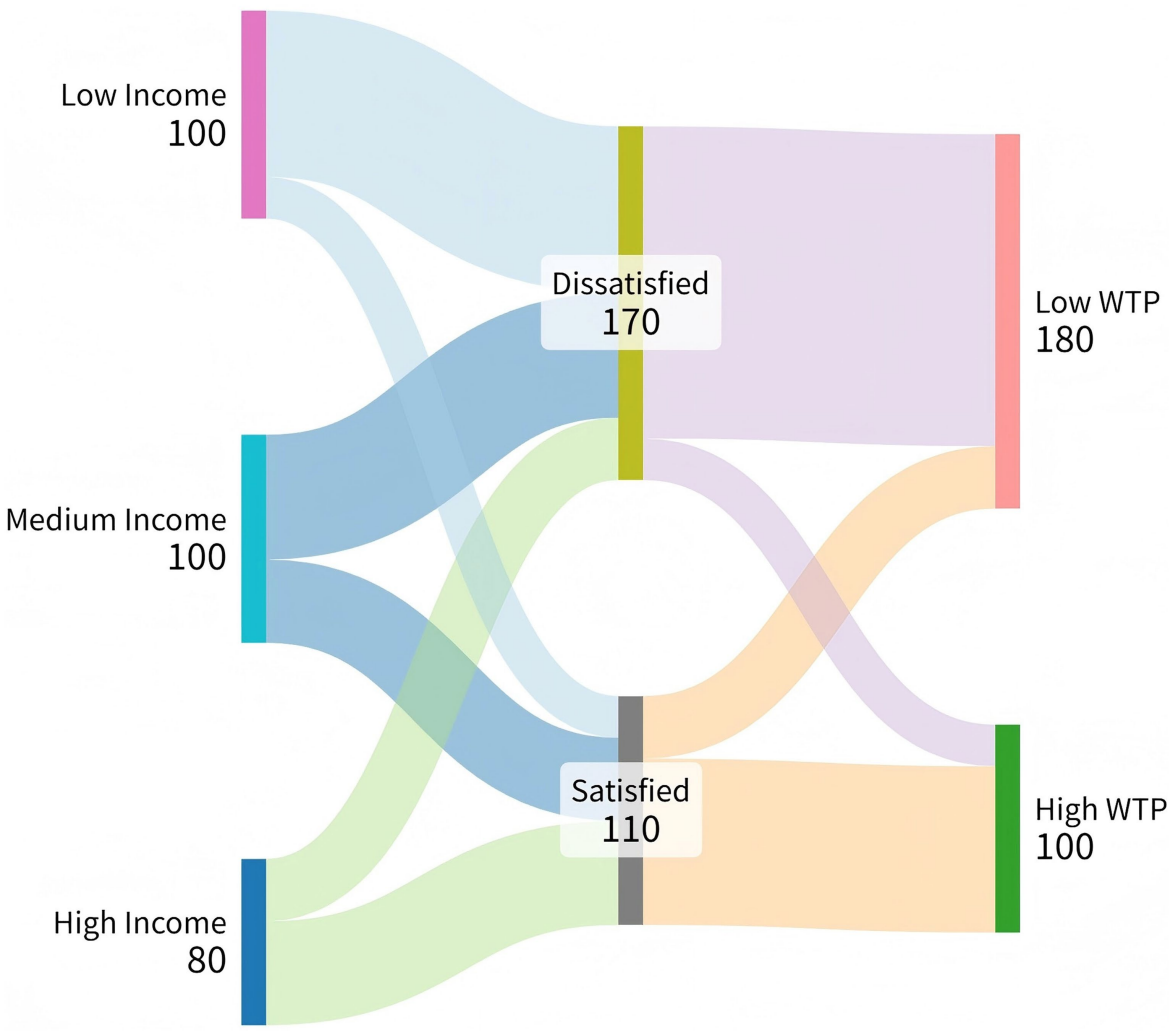
Sankey diagram illustrating the path from Income to Willingness-to-Pay. The flow demonstrates that regardless of income level, low service satisfaction predominantly leads to low willingness-to-pay, highlighting service quality as the key economic driver.

To further examine factors associated with workforce engagement (supply side) and payment-related patterns (demand side), additional analyses were conducted. On the supply side, multivariable binary logistic regression was used to identify factors associated with nursing students’ intention to work in SEC (Figure 4). Receipt of SEC-related educational intervention was significantly associated with higher intention to enter the field (OR = 2.85, 95% CI 1.45–5.60), indicating higher odds among students who had completed relevant coursework. Internship experience was not statistically significant in the model. On the demand side, a Sankey diagram was used to visualize transitions from income level to service satisfaction and willingness-to-pay (WTP) among older adults (Figure 5). Across income strata, a prominent flow was observed from “Not satisfied” to “Low WTP,” suggesting that lower satisfaction was associated with lower willingness to pay and that income level alone did not account for payment willingness. Notably, even among higher-income participants, dissatisfaction was frequently accompanied by low WTP, highlighting the potential importance of perceived service quality and alignment with user needs.

## 4 Discussion

### 4.1 The paradox of “illusory competence”: bridging the cognitive-digital divide

This study identified clear differences in SEC-related knowledge and privacy attitudes between nursing students and older adults. Nursing students reported higher self-perceived SEC knowledge, but this may not directly translate into the practical competencies needed to support older adults in community settings. The findings suggest that students’ familiarity with digital health concepts may exceed their understanding of older adults’ day-to-day experiences and needs. This is consistent with previous work (32) indicating that nursing students may report high confidence in using digital technologies while demonstrating comparatively limited competence in geriatric care. If such gaps are not addressed, SEC initiatives may risk focusing on technically advanced functions that are less acceptable or less relevant to end-users (33). These results highlight the need for nursing education to integrate digital health training with geriatric care competencies, communication skills, and user-centered approaches, rather than emphasizing technical knowledge alone (34).

### 4.2 Deconstructing the biomedical gaze: from surveillance to support

Differences in service priorities were observed, with nursing students placing greater emphasis on medical monitoring and older adults placing greater emphasis on daily life assistance. Nursing education is often grounded in acute-care contexts, which may lead students to associate smart care primarily with remote patient monitoring (35). Prior literature has also noted that continuous monitoring in home settings can raise concerns about privacy and the preservation of everyday normalcy (36). For community-dwelling older adults, the goal of aging in place may not align with technology use that resembles intensive clinical supervision. Monitoring functions that are not accompanied by support for activities of daily living (ADLs) and emotional well-being may be perceived as less acceptable. These findings suggest that SEC should be designed and delivered to support independence and daily functioning, rather than focusing solely on surveillance-oriented functions (37).

### 4.3 The “holistic care” deficit: reclaiming the human element in digital health

A critical insight from this study is the prioritization gap regarding psychosocial support. Although nursing care emphasizes holistic principles, an overemphasis on technical functions may reduce attention to psychosocial needs such as loneliness and emotional well-being (38). In this study, older adults placed greater importance on emotional support, indicating that psychosocial needs should be considered alongside physical safety. Previous reviews have noted that non-clinical and socially oriented components may be underrepresented in some digital health strategies for older adults (39). If smart care systems focus primarily on physical safety and monitoring while providing limited support for social connection, they may be less responsive to older adults’ broader needs. Consistent with prior work, technology should be designed to support, rather than replace, human interaction and social engagement (40). In practice, nurses can help integrate these tools into care in ways that facilitate communication and meaningful social connection (41).

### 4.4 Navigating the privacy paradox: trust as a clinical outcome

Differences in privacy attitudes suggest that trust is an important factor in SEC implementation. Nursing students reported lower levels of concern about data security than older adults (42). Although some studies have suggested that privacy concerns may be decreasing among more recent cohorts of older adults, continuous or 24/7 monitoring can still raise substantial privacy concerns for many users (43). If providers interpret these concerns primarily as reluctance to adopt new technologies, this may negatively affect communication and the care relationship. Therefore, privacy should be addressed as part of SEC implementation, including clear consent processes and user choice over data collection and monitoring functions. Approaches that provide users with more control, such as the option to pause or limit monitoring in specific situations, may help improve acceptability (44).

### 4.5 Drivers of workforce and market viability: beyond curriculum and income

Finally, our analysis of workforce and economic drivers highlights two implications for practice and policy. First, regarding workforce supply, the finding that educational intervention showed a stronger association with career intention than clinical internship suggests that current clinical placements may provide limited exposure to SEC-related practice. Clinical training could be strengthened by incorporating content on digital ethics and practical applications of smart care, with an emphasis on communication and user-centered care (45). Second, regarding market viability, the pattern that lower service satisfaction was associated with lower willingness-to-pay across income groups suggests that perceived service quality is a key factor in economic feasibility. For policymakers and developers, this indicates that improving service design and delivery to better align with older adults’ needs may be important for uptake and sustainability.

### 4.6 Limitations

This study has several limitations. First, the cross-sectional design precludes causal inference between SEC-related perceptions and associated factors. Second, participants were recruited from a single municipality in China using convenience sampling, which may limit the generalizability of the findings to other regions and healthcare contexts. Third, different data collection modes were used (face-to-face interviews for older adults and an online survey for nursing students). Although this approach improved feasibility given differences in digital literacy, it may have introduced mode-related bias, including potential social desirability effects in the interview-based responses. Fourth, age was collected in categories to protect privacy; therefore, the reported mean ages are estimates based on the midpoint method rather than exact calculations. Fifth, comparisons between nursing students and older adults should be interpreted with caution because the two groups differ substantially in age, educational background, and social roles. The observed differences may partly reflect these structural characteristics rather than solely SEC-related perceptions. Future studies could consider matching or adjustment strategies (e.g., propensity score methods), qualitative exploration, and multi-site sampling to improve comparability and external validity. Finally, longitudinal studies are needed to assess how nursing students’ perceptions change after targeted SEC-related education and community-based or smart-care clinical placements.

## 5 Conclusion

This study identified differences between nursing students and community-dwelling older adults in self-perceived SEC knowledge, privacy concerns, and service preferences. Nursing students reported higher perceived knowledge but lower privacy concerns than older adults, and their service preferences were more concentrated on medical and monitoring-related domains, whereas older adults placed greater emphasis on daily-life assistance and emotional support. These findings suggest that current educational exposure may not sufficiently prepare students to incorporate end-users’ privacy expectations and non-medical needs when considering SEC in community settings.

In terms of practice implications, nursing curricula may benefit from incorporating more community-based learning experiences and content related to digital ethics and privacy protection, so that students can better understand older adults’ perspectives and communicate SEC options appropriately. From a service development perspective, improving service quality and user experience, including privacy safeguards and usability, may be important for increasing acceptance. In addition, the economic pathway analysis indicates that service satisfaction is closely related to willingness-to-pay; therefore, improving satisfaction may be necessary to enhance uptake and sustainability of SEC services.

## Data Availability

The original contributions presented in the study are included in the article/Supplementary material, further inquiries can be directed to the corresponding author.
