# Supplementary material for: Illusory competence vs. real needs a cross-sectional study on the mismatch in Smart Senior Care (SSC) perceptions between nursing students and older adults
Source: Front Public Health. 2026 Apr 2;14:1777839. doi: 10.3389/fpubh.2026.1777839 (PMC13085051; doi:10.3389/fpubh.2026.1777839)
Supplement: Supplementary file 1 [file Supplementary_file_1.docx]

**Supplementary File 1**

**Part A: Questionnaire for Older Adults**

**Title:** Survey on Smart Elderly Care Community Construction and Innovation in Digital Inclusion Services

**Dear Resident:**
Hello! Thank you very much for taking the time to participate in this survey. This questionnaire aims to deeply understand the needs of residents in Shapingba District for elderly care services, their perception of smart elderly care communities, and their satisfaction and acceptance of digital inclusion services. Your feedback will serve as an important reference for promoting the development of related services. Please answer truthfully based on your actual situation. This questionnaire is anonymous, and all data will be used for statistical analysis only. Please feel free to answer with confidence.

**I. Personal Information**

**1. Your Age:** [Single Choice] *
○ A. Under 60 years old
○ B. 60 - 69 years old
○ C. 70 - 79 years old
○ D. 80 years old and above

**2. Your Gender:** [Single Choice] *
○ A. Male
○ B. Female

**3. Your Living Arrangement:** [Single Choice] *
○ A. Living alone
○ B. Living with spouse
○ C. Living with children
○ D. Living with other relatives
○ E. Elderly care institution

**4. Personal Monthly Disposable Income (RMB):** [Single Choice] *
○ A. 3,000 Yuan and below
○ B. 3,001 - 5,000 Yuan
○ C. 5,001 - 8,000 Yuan
○ D. Above 8,000 Yuan
○ E. No income source

**5. Your Occupation (Former or Current):** [Single Choice] *
○ A. Retired
○ B. Employee of enterprises/public institutions
○ C. Self-employed
○ D. Freelancer
○ E. Unemployed
○ F. Other

**6. Your Health Status:** [Single Choice] *
○ A. Good, no chronic diseases (e.g., diabetes, hypertension, coronary heart disease)
○ B. Good, have chronic diseases but controlled and stable
○ C. Fair, have chronic diseases requiring regular treatment
○ D. Poor, partially dependent in daily living
○ E. Very poor, completely dependent in daily living

**II. Current Status and Needs of Community Elderly Care**

**7. How well do you know the elderly care services currently provided by your community?** [Single Choice] *
○ A. Very well
○ B. Moderately well
○ C. A little
○ D. Almost nothing

**8. Your requirement for the professionalism of community elderly care services:** [Single Choice] *
○ A. Very high; requires professional and experienced medical staff
○ B. High; requires professionally trained personnel
○ C. General; basic services are sufficient
○ D. Do not pay attention to professionalism

**9. Your expected mode of community elderly care services (Multiple Choice):** *
□ A. Home-based care services
□ B. Centralized services at community centers
□ C. Home sickbeds (Hospital-at-Home)
□ D. Remote guidance
□ E. Other (Please specify): _________________

**10. Your degree of need for incorporating smart elements (e.g., health data monitoring, smart reminders) into community care services:** [Single Choice] *
○ Very much needed
○ Needed
○ Neutral
○ Not needed
○ Not needed at all

**11. Are you willing to authorize smart devices to collect health data for personalized care services?** [Single Choice] *
○ Very willing
○ Willing
○ Neutral
○ Unwilling
○ Very unwilling

**12. Are you willing to participate in training for using smart devices?** [Single Choice] *
○ Very willing
○ Willing
○ Neutral
○ Unwilling
○ Very unwilling

**13. Which aspects of elderly life do you think smart care services need to focus on most? (Multiple Choice):** *
□ A. Disease management (chronic disease care and guidance, etc.)
□ B. Daily living assistance (shopping, cooking, cleaning, etc.)
□ C. Psychological counseling and support
□ D. Physical exercise
□ E. Cultural and entertainment activities
□ F. Safety assurance (emergency rescue, fall prevention, etc.)
□ G. Other (Please specify): _________________

**14. Do you wish to participate in the design or decision-making process of community care services?** [Single Choice] *
○ A. Very much; willing to participate actively
○ B. Somewhat; will participate if time permits
○ C. Indifferent; as long as the service is good
○ D. Not really; find it troublesome
○ E. Not at all; trust the judgment of professionals

**15. Your satisfaction with the current elderly care services provided by the community:** [Single Choice] *
○ A. Very satisfied
○ B. Satisfied
○ C. Neutral
○ D. Dissatisfied
○ E. Very dissatisfied

**III. Cognition and Acceptance of Smart Elderly Care and Digital Inclusion Services**

**16. Your understanding of the concept of "Smart Elderly Care Community":** [Single Choice] *
○ A. Very well
○ B. Partially
○ C. Not at all

**17. Your main sources of information regarding smart elderly care services (Multiple Choice):** *
□ A. Community publicity
□ B. TV and radio
□ C. Newspapers and magazines
□ D. Online media (websites, social media, etc.)
□ E. Word of mouth (recommendations from friends/family)
□ F. Other (Please specify): _________________

**18. The potential impact of smart elderly care community construction on your life:** [Single Choice] *
○ A. Significant impact; will greatly improve life
○ B. Some positive impact
○ C. No impact
○ D. Uncertain (do not understand smart elderly care communities)

**19. Your degree of concern regarding privacy when smart devices collect personal data:** [Single Choice] *
○ A. Very concerned
○ B. Concerned
○ C. Not concerned

**20. Your evaluation of the operational difficulty of current smart devices (e.g., smart bracelets, emergency call devices):** [Single Choice] *
○ A. Easy to operate
○ B. Average; acceptable
○ C. Complex; need help
○ D. Never used

**21. Your expected functions of smart elderly care services (Multiple Choice):** *
□ A. Remote medical consultation
□ B. Remote rehabilitation training guidance
□ C. Health data monitoring and analysis
□ D. Daily life reminders (medication, activity reminders, etc.)
□ E. Online participation in cultural/entertainment activities
□ F. Social interaction platforms
□ G. Other (Please specify): _________________

**22. How would you like the community to help you better use smart care services? (Multiple Choice):** *
□ A. One-on-one training
□ B. Group training courses
□ C. Detailed operation manuals or video tutorials
□ D. Volunteer assistance
□ E. Family member assistance
□ F. Other (Please specify): _________________

**23. Your evaluation of the professional level and service enthusiasm of community care personnel:** [Single Choice] *
○ Very high
○ High
○ Average
○ Poor
○ Very poor

**24. Your evaluation of the convenience and timeliness of information updates (health guides, community activity promotion):** [Single Choice] *
○ Very high
○ High
○ Average
○ Poor
○ Very poor

**25. Your evaluation of the emergency response capability and the supervision/feedback mechanism of community care services:** [Single Choice] *
○ Very strong
○ Strong
○ Average
○ Weak
○ Very weak

**26. In which aspects do you think community elderly care services need improvement? Please briefly describe your ideal solution. (Multiple Choice):** *
□ A. Improving the advancement and sufficiency of facilities and equipment
□ B. Improving the professional level and attitude of service personnel
□ C. Enhancing service convenience and accessibility (e.g., shortening response time, expanding coverage)
□ D. Increasing the degree of personalization
□ E. Reasonably adjusting service fees
□ F. Strengthening timely information updates and releases
□ G. Improving emergency response capabilities
□ H. Perfecting supervision and feedback mechanisms
□ I. Strengthening cooperation with medical institutions
□ J. Other (Please specify): _________________
□ Suggested Solution: _________________

**27. Your acceptable monthly fee range for paid smart elderly care services (RMB):** [Single Choice] *
○ A. Below 300 Yuan
○ B. 300 - 500 Yuan
○ C. 501 - 1,000 Yuan
○ D. Above 1,000 Yuan
○ E. Do not accept paid services

**IV. Policy Support and Suggestions**

**28. Are you aware of government policies regarding the construction of smart elderly care communities?** [Single Choice] *
○ A. Very aware
○ B. Partially aware
○ C. Not aware

**29. Your opinion on the implementation effect of existing smart elderly care community policies:** [Single Choice] *
○ A. Very good; policies are well implemented
○ B. Some effectiveness
○ C. Implementation needs strengthening
○ D. No effect at all

**30. What do you think are the main responsibilities of the community in smart elderly care services? (Multiple Choice):** *
□ A. Providing basic care facilities and equipment
□ B. Organizing care knowledge publicity and education activities
□ C. Coordinating resources from all parties to provide comprehensive services
□ D. Supervising service quality and protecting residents' rights
□ E. Collecting resident feedback and continuously improving services
□ F. Other (Please specify): _________________

**31. Your evaluation of the mechanism for resident participation in decision-making during smart elderly care community construction:** [Single Choice] *
○ A. Very perfect; residents can fully express their opinions
○ B. Basically perfect; there are certain channels for participation
○ C. General; participation is limited
○ D. Not very perfect; lack of effective participation channels
○ E. No opportunity for participation

**32. Your evaluation of the collaboration effect between medical and social sectors in smart elderly care community construction:** [Single Choice] *
○ A. Very effective; seamless cooperation between departments
○ B. Basically effective; but needs strengthening
○ C. Average; coordination difficulties exist
○ D. Poor; lack of synergy
○ E. Almost no cross-departmental collaboration

**33. Do you support the government introducing social capital to participate in smart elderly care community construction through the Public-Private Partnership (PPP) model?** [Single Choice] *
○ A. Support
○ B. Do not support
○ C. Indifferent

**34. Which policies do you think can effectively promote residents' participation and use of related services? (Multiple Choice):** *
□ A. Service fee subsidy policies (e.g., government purchasing services)
□ B. Health data management preferential policies (e.g., free physical exams, data security insurance)
□ C. Resident training and education support policies (e.g., smart device usage courses)
□ D. Service experience activity policies (e.g., free trial of smart devices)
□ E. Community point incentive policies (e.g., exchanging points for services)
□ F. Other (Please specify): _________________

**35. Your overall expectations and suggestions for the construction of smart elderly care communities:** [Open-ended]

**Part B: Questionnaire for Nursing Students**

**Title:** Survey on Smart Elderly Care Communities (Nursing Students/Practitioners)

**Dear Nursing Colleagues/Students:**
Hello! We are conducting a survey on the understanding of smart elderly care communities among nursing students and practitioners. The aim is to understand the current level of knowledge and willingness to participate in community health and elderly care. This questionnaire is anonymous, and all data will be used for statistical analysis only. We will strictly keep your personal information confidential. Please answer truthfully based on your actual situation. Your answers are crucial to our research. Thank you for your support and cooperation!

**I. Basic Information**

**1. Your Academic Year:** [Single Choice] *
○ A. First Year (Freshman)
○ B. Second Year (Sophomore)
○ C. Third Year (Junior)
○ D. Fourth Year (Senior) and above

**2. Do you have any internship experience in elderly care institutions or communities?** [Single Choice] *
○ A. Yes
○ B. No

**3. Does your school offer courses related to smart elderly care?** [Single Choice] *
○ A. Yes
○ B. No

**II. Cognition of Smart Elderly Care**

**4. How well do you understand the definition of a smart elderly care community?** [Single Choice] *
○ A. Very well
○ B. Moderately well
○ C. Average
○ D. Not very well
○ E. Not at all

**5. Do you know which smart devices or technologies are usually included in a smart elderly care community? (Multiple Choice):** *
□ A. Remote health monitoring equipment
□ B. Intelligent nursing robots
□ C. Health management information systems
□ D. Emergency call systems
□ E. Other (Please specify): _________________

**6. Do you know what the main service contents of a smart elderly care community are? (Multiple Choice):** *
□ A. Health assessment and monitoring
□ B. Chronic disease management
□ C. Rehabilitation nursing
□ D. Daily life care
□ E. Spiritual/Emotional support
□ F. Other (Please specify): _________________

**7. Are you clear about the differences between smart elderly care communities and traditional elderly care communities?** [Single Choice] *
○ A. Very clear
○ B. Relatively clear
○ C. Average
○ D. Not very clear
○ E. Completely unclear

**8. How well do you know the policy support and industry standards for smart elderly care communities?** [Single Choice] *
○ A. Very well
○ B. Moderately well
○ C. Average
○ D. Not very well
○ E. Not at all

**III. Beliefs regarding Smart Elderly Care**

**9. What do you think of the development prospects of smart elderly care communities?** [Single Choice] *
○ A. Very good
○ B. Good
○ C. Average
○ D. Not very good
○ E. Very bad

**10. Do you believe that smart elderly care technology can improve the quality of life for the elderly?** [Single Choice] *
○ A. Strongly believe
○ B. Believe
○ C. Neutral
○ D. Do not really believe
○ E. Do not believe at all

**11. Do you think it is necessary for nursing students to master knowledge and skills related to smart elderly care?** [Single Choice] *
○ A. Very necessary
○ B. Necessary
○ C. Neutral
○ D. Not very necessary
○ E. Completely unnecessary

**12. Are you confident about the safety and privacy protection of smart elderly care communities?** [Single Choice] *
○ A. Very confident
○ B. Confident
○ C. Neutral
○ D. Not very confident
○ E. Not confident at all

**13. Do you think smart elderly care communities can effectively alleviate the current pressure on elderly care services?** [Single Choice] *
○ A. Very effective
○ B. Effective
○ C. Neutral
○ D. Not very effective
○ E. Completely ineffective

**IV. Initiative in Participation**

**14. Have you actively studied knowledge related to smart elderly care?** [Single Choice] *
○ A. Often actively study
○ B. Occasionally actively study
○ C. Rarely actively study
○ D. Never actively study

**15. Have you attended lectures, training, or practical activities related to smart elderly care communities?** [Single Choice] *
○ A. Attended multiple times
○ B. Attended 1-2 times
○ C. Never attended

**16. Have you come into contact with smart elderly care devices or technologies during internships or practice?** [Single Choice] *
○ A. Often contact
○ B. Occasionally contact
○ C. Rarely contact
○ D. Never contacted

**17. Are you willing to engage in nursing-related work in a smart elderly care community in the future?** [Single Choice] *
○ A. Very willing
○ B. Willing
○ C. Indifferent
○ D. Unwilling
○ E. Very unwilling

**18. Would you recommend the smart elderly care community model to others?** [Single Choice] *
○ A. Definitely yes
○ B. Probably yes
○ C. Uncertain
○ D. Probably not
○ E. Definitely not

**Thank you again for your participation!**
